# Attenuation of PTEN increases p21 stability and cytosolic localization in kidney cancer cells: a potential mechanism of apoptosis resistance

**DOI:** 10.1186/1476-4598-6-16

**Published:** 2007-02-14

**Authors:** Pei-Yin Lin, Susan P Fosmire, See-Hyoung Park, Jin-Young Park, Shairaz Baksh, Jaime F Modiano, Robert H Weiss

**Affiliations:** 1 Division of Nephrology, Department of Internal Medicine, University of California, Davis, CA, USA; 2 Integrated Department of Immunology, University of Colorado at Denver and Health Sciences Center, Denver, CO, USA; 3 Department of Pediatrics, University of Alberta, Edmonton, T6G 2H7, AB, Canada,; 4 University of Colorado Cancer Center, Aurora, CO, USA; 5 Department of Veterans' Affairs Northern California Health Care System, Sacramento, CA, USA

## Abstract

**Background:**

The *PTEN *(Phosphatase and Tensin homolog deleted on chromosome Ten) tumor suppressor gene is frequently mutated or deleted in a wide variety of solid tumors, and these cancers are generally more aggressive and difficult to treat than those possessing wild type *PTEN*. While PTEN lies upstream of the phosphoinositide-3 kinase signaling pathway, the mechanisms that mediate its effects on tumor survival remain incompletely understood. Renal cell carcinoma (RCC) is associated with frequent treatment failures (~90% in metastatic cases), and these tumors frequently contain PTEN abnormalities.

**Results:**

Using the ACHN cell line containing wild type PTEN, we generated a stable PTEN knockdown RCC cell line using RNA interference. We then used this PTEN knockdown cell line to show that PTEN attenuation increases resistance to cisplatin-induced apoptosis, a finding associated with increased levels of the cyclin kinase inhibitor p21. Elevated levels of p21 result from stabilization of the protein, and they are dependent on the activities of phosphoinositide-3 kinase and Akt. More specifically, the accumulation of p21 occurs preferentially in the cytosolic compartment, which likely contributes to both cell cycle progression and resistance to apoptosis.

**Conclusion:**

Since p21 regulates a decision point between repair and apoptosis after DNA damage, our data suggest that p21 plays a key role in mechanisms used by PTEN-deficient tumors to escape chemotherapy. This in turn raises the possibility to use p21 attenuators as chemotherapy sensitizers, an area under active continuing investigation in our laboratories.

## Background

The *PTEN *(Phosphatase and Tensin homolog deleted on chromosome Ten) gene encodes a dual lipid and tyrosine phosphatase that regulates signaling through the PI3K/Akt pathway [1], and acts as a tumor suppressor protein that is frequently mutated or deleted in human cancers. Studies have shown that mice heterozygous for *PTEN *develop spontaneous tumors[2,3], and that conditional tissue-specific tissue disruption of *PTEN *leads to tumors in the affected tissues[4,5]. Through its actions on multiple downstream signaling proteins, including but not limited to the PI3K/Akt pathway, PTEN has the capacity to affect a variety of cancer-relevant signaling cascades.

Germline mutations of *PTEN *occur in 80% of patients with Cowden syndrome, which is characterized by the occurrence of multiple non-cancerous hamartomas; in addition, these patients are at high risk for breast, thyroid, and endometrial carcinomas, as well as an increased risk of bladder and renal cell carcinoma (RCC)[6]. Consistent with these data, PTEN protein and gene expression have been variously described as reduced[7,8], absent[9], mutated[10], or deleted [11] in human RCCs; a recent study demonstrated PTEN loss in 20% of RCCs[12] and another study quoted an LOH of 27% in kidney cancer[13]. Since RCC is a malignancy associated with frequent treatment failures when metastatic, and because RCC and other tumors lacking PTEN are often resistant to conventional chemotherapy[14,15], the mechanism by which PTEN contributes to chemotherapy failure is of immediate clinical importance and may lead to new therapeutic options for patients with such cancers.

Cell cycle progression, both in normal and cancer cells, is finely regulated by the interplay between the cyclins, cyclin-dependent kinases (CDKs) and CDK inhibitors (CKIs), as well as by fluctuation in their levels at different points of the cell cycle (reviewed in [16]). The earliest described role of p21 was in cyclin/cdk inhibition[17,18], but more recent data also has shown that p21 is involved in positive effects on cyclin/cdk activation[17,19,20] through its "assembly factor" function[21]. In addition, p21 has been shown to be anti-apoptotic in many tissues, including cancer [22,23], and, as such, has been suggested to be a target for cancer therapy[24]. There are also reports of a role of p21 in inducing senescence, a mechanism which seems to protect against malignant transformation[25]. We have previously shown that p21 is a prognostic marker in clear cell RCC (ccRCC) such that its elevated levels portend a poorer prognosis in patients who have metastatic ccRCC at diagnosis[26,27].

While *p21 *is transcriptionally regulated by p53[28] (hence its function in DNA damage repair), the mechanisms that regulate the activity of p21 and its post-translational modification are less clear. A previous report demonstrated that p21 is phosphorylated by Akt, which leads to increased p21 stability as well as enhanced cell survival[29], and another report showed that cytoplasmic localization of p21 results from HER2/Neu activation of Akt with subsequent p21 phosphorylation[30]. We have shown that p21 accumulates in the cytoplasm of actively growing cells [31] and that forced localization of p21 to the cytosolic compartment results in increased cell growth[32] and resistance to apoptosis [33]. Given the complex relationship between PTEN, phosphoinositide-3 kinase (PI3K), Akt, and p21, which are all signaling proteins involved in cell growth and apoptosis in cancer, we now address how PTEN deficiency influences p21. In this study, we demonstrate that, in an RCC cell line that retains wild type genes for PTEN and p53, knockdown of PTEN using RNA inhibition increases p21 stability, but not transcription. This event results in increased cytosolic localization of this protein, a property which, having been earlier shown to result in apoptosis resistance and cell cycle progression, may explain the chemoresistance of PTEN-deleted tumors.

## Results

### Rapamycin decreases p21 in response to cisplatin-induced DNA damage

Inhibition of mTOR has been shown to decrease p21 in human non-small cell lung carcinoma cells, and, in fact, such attenuation of p21 is required for sensitization of these cells to apoptosis induced by DNA damage [34]. A clinical trial using the rapamycin analog CCI-779 (an mTOR inhibitor) showed this compound has antitumor activity against RCC[35]. We thus hypothesized that both the results from this trial and the chemotherapy resistance that is characteristic of PTEN-deficient RCC may be due, at least in part, to the anti-apoptotic function of p21.

**Figure 1:**
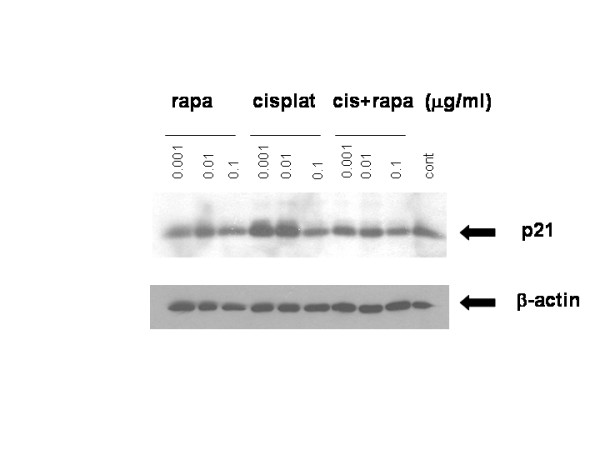
**Cisplatin increases p21 in ACHN cells**. ACHN cells were incubated with cisplatin and/or rapamycin for 24 h at the indicated concentrations and lysates were immunoblotted for p21. β-actin expression was used as a loading control. A representative experiment is shown of three done.

There is no "standard" chemotherapeutic agent in RCC, since none has been proven unequivocally effective in this disease [36] which is highly resistant to chemotherapy[37]. However, for these studies we have utilized the DNA-damaging agent cisplatin, given the available data on sensitization of tumor cells to this agent with p21 attenuation by the mTOR inhibitors and other agents[24,34]. To begin to address the hypothesis that the chemotherapy resistance of PTEN-deficient RCC may be due to p21 induction, we asked whether cisplatin increases p21 in ACHN cells (a PTEN-wt ccRCC cell line) and whether this increase is antagonized by mTOR inhibition (which has been shown to have antitumor activity). When ACHN cells were incubated with cisplatin at concentrations of 0.001 and 0.01 μg/ml, p21 levels increased, presumably in response to DNA damage; yet, this increase did not occur in cells incubated with rapamycin (Fig. 1). In addition, when used in combination with cisplatin, rapamycin inhibited the accumulation of p21 (Fig. 1). Thus, DNA damage leads to increased levels of p21, and this increase is prevented by inhibition of mTOR.

### Knockdown of PTEN increases steady-state levels of p21 and causes resistance to DNA-damage induced apoptosis

**Figure 2:**
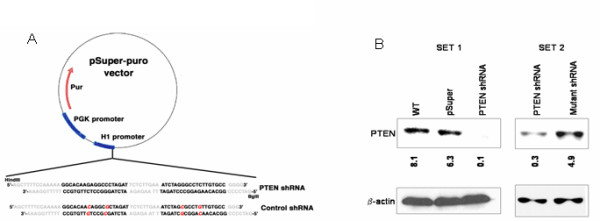
**Attenuation of PTEN expression by pSuper RNAi system**. a. Illustration of pSuper RNAi construct with mutations (in red) in the control shRNA. b. Effect of shRNA on PTEN expression in ACHN cells in two separate experiments.

To determine whether the chemotherapy resistance of PTEN-altered RCCs may relate to p21, we generated two PTEN-knockdown ccRCC cell lines using RNA interference. We utilized an shRNA construct to stably attenuate PTEN expression in the PTEN-wt RCC cell line, ACHN (Fig. 2a). Stable clones of PTEN shRNA (N = 2)-, mutant-shRNA- (Fig. 2a) and empty vector-transfected (control) cells were selected and examined for expression of PTEN and p21. As expected, levels of PTEN were significantly reduced (by as much as 20-fold) in cells transfected with PTEN shRNA, but not in those containing pSuper (empty) vector alone or mutant shRNA (Fig. 2b), indicating that the attenuation of PTEN was not due to non-specific effects of introducing shRNA into the cells.

**Figure 3:**
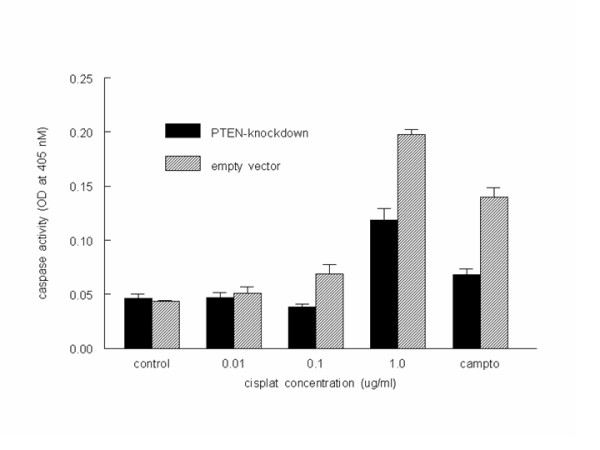
**PTEN-knockdown reduces sensitivity to cisplatin-induced apoptosis**. ACHN cells stably transfected with PTEN-shRNA (black bars) or pSuper empty vector control (gray bars) were incubated with the indicated concentrations of cisplatin for 24 h. Caspase-3 activity was measured as an indicator of apoptosis as described in Materials and Methods.

**Figure 4:**
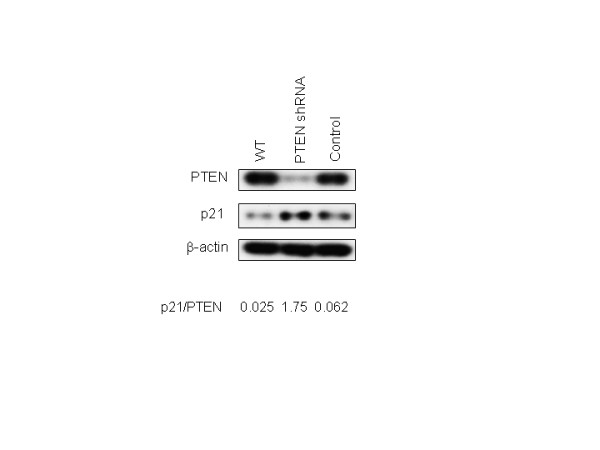
**p21 is increased in PTEN-knockdown cells**. ACHN cells stably transfected with PTEN-shRNA or empty vector control were immunoblotted with PTEN, p21, and β-actin. Quantification of p21 and PTEN bands was determined by densitometry, normalized to β-actin expression, and expressed as fold-change p21/PTEN.

To determine whether PTEN-knockdown cells were associated with apoptosis resistance, we incubated these cells with various concentrations of cisplatin as well as with the known apoptosis inducer camptothecin. At cisplatin concentrations of 0.1 and 1.0 μg/ml, as well as with incubation of camptothecin at 4 μM, the PTEN-knockdown cells were resistant to apoptosis measured by total Caspase activity (Fig. 3). Intriguingly, p21 levels were increased by ~5-fold in the PTEN-knockdown cells (Fig. 4) suggesting that p21 is involved in the resistance to apoptosis seen upon loss or inactivation of PTEN; subsequent experiments were performed to ascertain the mechanisms underlying this effect.

**Figure 5:**
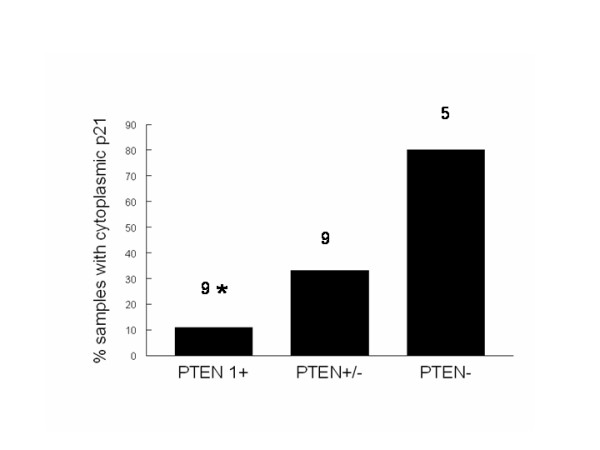
**Inverse relationship between expression of PTEN and cytoplasmic localization of p21 in canine melanoma**. A series of naturally occurring canine melanomas on which we previously reported expression of PTEN and p21 (see text) was re-examined to determine if there was a consistent relationship between expression of these proteins. Data were evaluated from tumors that had easily detectable PTEN staining in >30% of cells (1+, N = 9), weak expression of PTEN (required high magnification for confirmation) in >30% of cells (+/-, N = 9), or no expression of PTEN (-, N = 5). Each tumor was then examined for p21 intensity and subcellular localization. Data represent the % of samples that showed cytoplasmic p21 for each group. The numbers over each bar represent the sample size for each group; *p < 0.03 compared to PTEN(-).

In order to demonstrate universality of the PTEN/p21 relationship, we re-examined a series of naturally occurring canine melanomas on which we had previously reported expression or tumor suppressor and cell cycle proteins[38]. Semi-quantitative assessment of p21 and PTEN using immunohistochemistry showed an inversely proportional relationship between expression of PTEN and cytoplasmic localization of p21 (Fig. 5), an indirect indicator of increased expression (see below). These data are consistent with what we observed when we knocked down PTEN in RCC cells *in vitro*.

### Stability of p21 is augmented in PTEN-knockdown cells

**Figure 6:**
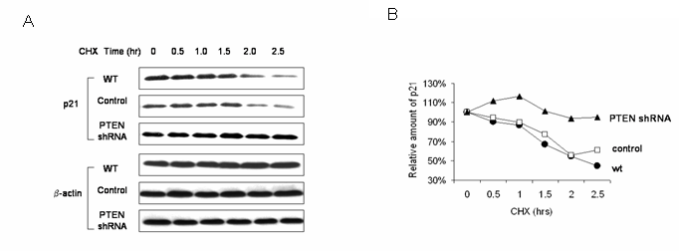
**The stability of p21 is increased by PTEN-knockdown**. a. Wild type ACHN cells as well as those stablytransfected with PTEN-shRNA or empty vector control were incubated with cycloheximide (CHX; 20 μg/ml) for the times indicated and immunoblotted with antibodies against p21 and β-actin. b. Graphical representation of the data from densitometric quantification of p21 and β-actin reported as ratio of p21/β-actin and normalized to the "0 CHX control" for each lane. A representative experiment of four done is shown.

In light of previous reports that p21 is phosphorylated by Akt, a kinase which lies downstream of both PI3K and PTEN, and that phosphorylation of p21 is associated with its increased stability [29,30,39], we next asked whether p21 stability is altered in PTEN-downregulated cells. Since initial efforts showed no change in p21 mRNA in PTEN knockdown as compared to PTEN-wt cells (data not shown), we utilized the protein synthesis inhibitor cycloheximide (CHX) to assess protein stability. Incubation of the cells with CHX abolishes translation of new p21, such that tracking p21 levels over time can be used as an indicator of its half-life. We incubated ACHN cells with CHX and followed the disappearance of the protein by immunoblotting. Both wild type cells and cells containing empty pSuper vector as control showed the half-life of p21 was ~2 h, consistent with previous reports[40,41] (Fig. 6). However, in PTEN-knockdown cells, p21 degradation was markedly reduced, with minimal change in p21 protein levels after 2.5 h (Fig. 6).

**Figure 7:**
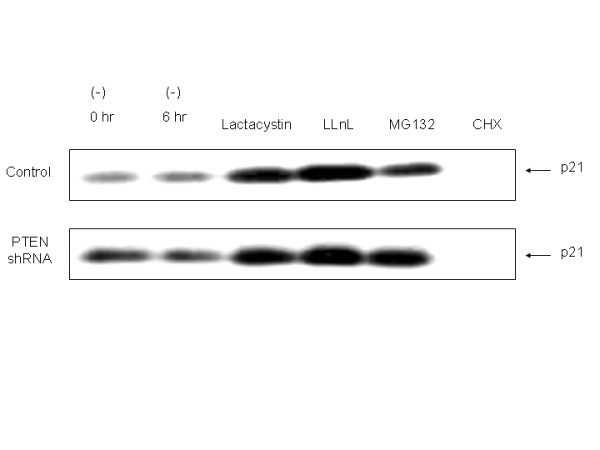
**PTEN knockdown does not alter proteasome-dependent degradation**. Cells stably transfected with PTEN-shRNA or empty vector were treated with three different proteasome inhibitors (lactastatin, 20 mM; LLnL, 50 mM; MG132, 1 mM) or cycloheximide (CHX; 20 μg/ml) for 6 h, followed by immunoblotting for p21. A representative experiment is shown of three done.

p21 levels are regulated by the ubiquitin/proteasome pathway [42], such that a potential mechanism of prolonged p21 stability involves inactivation of this system. Thus, we next examined whether the proteasome system itself was altered in PTEN-knockdown cells using three distinct proteasome inhibitors, lactastatin, N-acetyl-L-leucinyl-L-leucinal-L-norleucinal (LLnL), and MG132. We found a similar increase in p21 levels after proteasome inhibition, using each of the inhibitors, in both control transfectants and PTEN-knockdown cells (Fig. 7), suggesting that the proteasome pathway is intact in both cell lines. The cells incubated with CHX for 6 h showed minimal p21 due to the relatively short half-life of this protein (see Fig. 6), a fact which is not altered by the continuing presence of PTEN shRNA and its effects on PTEN.

### Inhibition of PI3K or Akt activation reverts p21 stability in PTEN-knockdown cells

**Figure 8:**
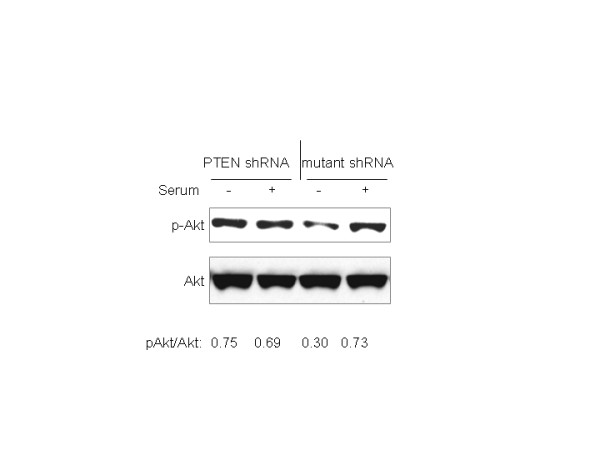
**Akt phosphorylation is increased in PTEN-knockdown cells**. Subconfluent ACHN cells stably transfected with PTEN or mutant control shRNA were transferred to serum-deficient media (0.5% FBS). After 18 hr, fresh serum-deficient media (0.5% serum, "-") or serum-replete media (10% serum, "+") was added for 30 minutes. Cell lysates were immunoblotted with antibodies against phosphorylated and total Akt. Lysates from 3T3 murine fibroblasts were used as controls. Densitometry of p-Akt is normalized to Akt.

While PTEN acts primarily on PI3K through its lipid phosphatase activity, it is conceivable that there is a direct effect of PTEN on p21 that is independent of the PI3K/Akt pathway, similar to the effects on PTEN on cellular migration that are mediated partly or exclusively through the C-terminal domains[43]. To investigate this possibility, we tested p21 stability under conditions where we inhibited the activity of either PI3K or Akt. First, we confirmed that loss of PTEN had the expected effects on activation of PI3K by evaluating Akt phosphorylation at Ser 473 (an activating event that requires PI3K activity). The levels of steady state Akt phosphorylation were high both under conditions of serum-deprivation and serum-stimulation in PTEN-knockdown cells (Fig. 8, lanes 1 and 2), indicating that attenuation of PTEN removed its antagonistic effects on PI3K-mediated Akt activation. Conversely, serum-deprivation led to a predictable reduction of phospho-Akt in cells transfected with the mutant control shRNA, yet Akt phosphorylation remained responsive to serum-stimulation in these cells (Figure 8, lanes 3 and 4), as is observed in wild type ACHN cells. This provides evidence of specificity, and excludes non-specific effects of shRNA transfection on PTEN attenuation. We also observed increased levels of phospho-Mdm2 in PTEN-knockdown cells (data not shown), which similarly supports a functional effect of PTEN-knockdown on related signaling pathways.

**Figure 9:**
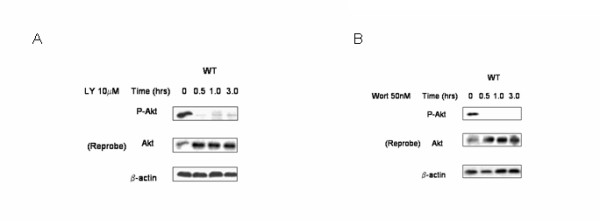
**Akt phosphorylation is reduced by PI3K inhibition**. Wild type ACHN cells were incubated with (a) LY-294,002 (10 μM) or (b) wortmannin (50 nM) for the times indicated and immunoblotted with antibodies against Akt, phospho-Akt, or β-actin. A representative experiments is shown of three done.

**Figure 10:**
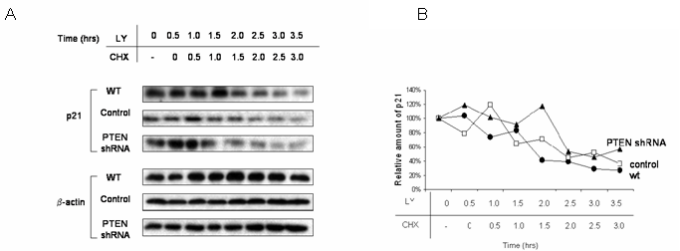
**Stabilization of p21 in PTEN-knockdown cells iseabrogated by PI3K inhibition with LY-294,002**. a. Wild type ACHN cells as well as those stably transfected with PTEN-shRNA or empty vector were incubated with LY-294,002 (10 μM) for the times indicated in the presence of cycloheximide (CHX; 20 μg/ml) for the times indicated, and cell lysate was immunoblotted with antibodies against p21 or β-actin. b. A graphical representation of the data using densitometry of p21 and actin bands is reported as ratio of p21/β-actin and is normalized to the "0 CHX control" for each lane. A representative experiment is shown of three done.

**Figure 11:**
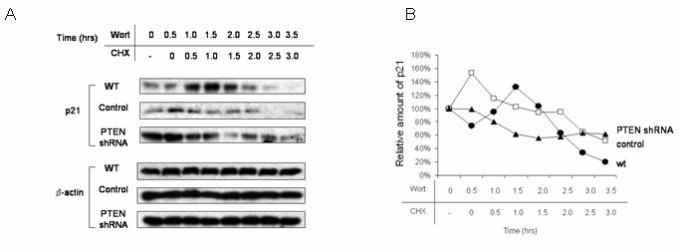
**Stabilization of p21 in PTEN-knockdown cells is abrogated by PI3K inhibition with wortmannin**. a. Wild type ACHN cells as well as those stably transfected with PTEN-shRNA or empty vector were incubated with wortmannin (50 nM) for the times indicated in the presence of cycloheximide (CHX; 20 μg/ml) for the times indicated, and cell lysates were immunoblotted with antibodies to p21 and β-actin. b. A graphical representation of the data using densitometry of p21 and actin bands is reported as ratio of p21/β-actin and is normalized to the "0 CHX control" for each lane. A representative experiment is shown of three done.

Next, we verified that the observed effects of PTEN inhibition on p21 stability were indeed mediated through induction of PI3K activity. Incubation of wild type cells with the selective PI3K antagonists LY-294,002 (10 μM) or wortmannin (50 nM) for 0.5 to 3 hr completely abolished Akt phosphorylation (Fig. 9). Examination of cells grown under the previously established conditions of CHX incubation, and also in the presence of either LY-294,002 (Fig. 10) or wortmannin (Fig. 11), restored the kinetics of p21 degradation in PTEN-attenuated cells to the levels seen in wild type cells and control transfectants; that is, the half life of this protein (2 – 2.5 h) was no longer significantly increased in the PTEN-attenuated cells than it was in the control cells, indicating that the augmentation of p21 stability in PTEN-knockdown cells was mediated through PI3K.

**Figure 12:**
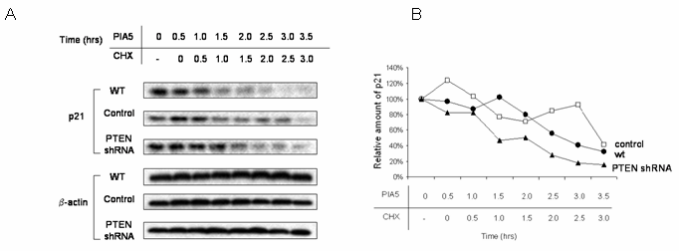
**Stabilization of p21 in PTEN-knockdown cells is abrogated by Akt inhibition**. a. Wild type ACHN cells as well as those stably transfected with PTEN-shPNA or empty vector were incubated with the phosphatidylinositol ether lipid analog PIA5 (10 μM), which prevents Akt activation, for the times indicated in the presence or absence of cycloheximide (CHX;20 μg/ml), and cell lysate was immunoblotted with antibodies against p21 and β-actin. b. A graphical representation of the data using densitometry of p21 and β-actin bands is reported as ratio of p21/actin and is normalized to the "0 CHX control" foreach lane. A representative experiment is shown of three done.

There is evidence to suggest that enhanced stability of p21 is caused by phosphorylation of p21 on Ser146 by Akt[29]. To determine if activated Akt was required for the stabilization of p21 upon PTEN attenuation, we utilized PIA5, one of a new class of phosphatidylinositol ether lipid analogues that prevent Akt activation and can selectively kill lung and breast cancer cells with elevated Akt activity[44]. When cells were incubated with PIA5 (10 μM) for 30 min prior to addition of CHX, we found that, as was seen with the PI3K inhibitors, the enhanced stability of p21 was abrogated and the half-life of the protein reverted to that seen in wild-type cells (Fig. 12). Thus, PTEN augments p21 stability via two downstream elements of the PI3K growth and survival signaling pathway, PI3K and Akt.

### Cytosolic localization of p21 is augmented in PTEN-downregulated cells

**Figure 13:**
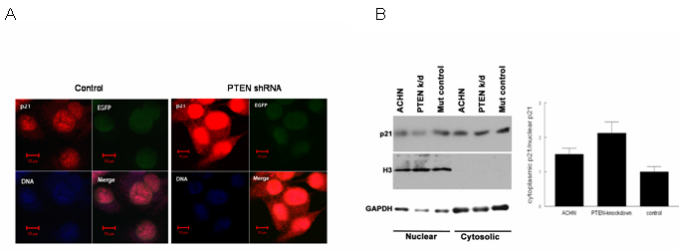
**PTEN attenuation enhances p21 expression and cytosolic localization**. a. ACHN cells stably transfected with PTEN-shRNA or empty vector were immunostained with anti-p21 antibody combined with Alexa Fluor^® ^594-conjugated secondary antibody for detection under UV microscopy. DAPI was used to demarcate nuclei, and EGFP (expressed by the plasmid) was used as a transfection control. b. Cells were fractionated for immunoblotting, with antibodies against mono methyl Histone H3-Lys4 and GAPDH used as nuclear and cytosolic controls, and p21 levels were quantified using densitometry. The ratio of cytosolic/nuclear p21 is shown graphically; the error bars indicate intra-experimental variation (standard deviation) from two replicates.

Subcellular localization of p21 is important in dictating its effect on cell growth and apoptosis, such that cytosolic p21 has been shown to result in activation of anti-apoptotic [33] and proliferative [30,32] machinery, resulting in more aggressive tumor behavior and a poorer patient prognosis[26,45,46]. We utilized immunofluorescence and immunohistochemistry techniques to assess subcellular distribution of p21 in PTEN-knockdown cells. Results were similar using both methods; i.e., p21 levels were substantially increased in the PTEN-knockdown cells as compared to control transfectants (Fig. 13a). Moreover, cytosolic localization of p21 also was increased in PTEN-knockdown cells (Fig. 12a).

To confirm this finding, we performed subcellular localization of PTEN-knockdown and mutant shRNA control ACHN cells to determine whether p21 is directed more to the cytosol as a result of PTEN attenuation. In agreement with the immunofluorescence data, we found that the ratio of p21 in the cytosol as compared to the nucleus was markedly enhanced in PTEN-KD cells (Fig. 13b). Whether these findings are due to a generalized increase in cellular p21 with a stoichiometric redistribution of a proportion of the total p21 from the nucleus to the cytosol, or to a specific augmentation of cytosolic p21 through a separate signaling mechanism (such as alteration of the nuclear localization sequence), remains to be determined. However, augmentation of total p21 as well as specific increases in cytosolic p21 in PTEN-deficient cells may be an important mechanism through which PTEN-altered tumors acquire resistance to chemotherapy[24].

## Discussion

Many malignancies, including kidney cancer, are characterized by resistance to DNA-damaging chemotherapy. While the mechanisms of this effect remain incompletely understood, they appear to relate to activation of survival pathways that occur in the setting of DNA damage repair. For example, exposure to ionizing radiation causes DNA strand breaks leading to increases in p53, which in turn activates either the cell death (apoptosis) program, or alternatively, genes involved in cell cycle arrest and subsequent DNA repair (reviewed in [24]). Thus, tumors harboring p53 mutations (~50% of all human malignancies), fail to apoptose or growth arrest after DNA damage[47,48] and may thus escape death upon exposure to chemotherapeutic agents; this can lead to chemotherapy failure. The cyclin kinase inhibitor p21 is induced by p53 in situations of DNA damage, and it likely plays a role in the decision pathways leading to apoptosis or DNA repair [24]; for this reason, not only has p21 been proposed as a target for chemotherapy sensitization, but also, the mechanisms by which p21 becomes activated have attracted considerable interest in the study of chemotherapy-resistant cancers.

p21 was initially characterized based on its function as an inhibitor of G1 cyclin kinases [17,18], but after the discovery that p21 possesses pro-proliferative[32] as well as anti-apoptotic [33] effects, research on this protein expanded to include to its roles in cancer progression and chemotherapy sensitivity. A putative relationship of p21 to PTEN was proposed after reports emerged that p21 is phosphorylated and stabilized by Akt[29,39]. Our present findings reported here, that PTEN deficiency augments p21 stability and alters its subcellular localization so that it is situated more in the cytosolic compartment, may explain at least part of the observed chemotherapy resistance that is observed in PTEN-deficient tumor cell lines as well as in tumors derived from PTEN-knockout mice[49].

Other investigators have demonstrated a tissue-specific relationship between PTEN and p21. Using a Cre-Lox model of tissue-specific PTEN-deficiency, Yoo et al detected higher levels of p21 protein and transcripts in PTEN-deficient bladder tissue (but not prostate), a finding which they interpreted as a potential bladder-specific tumor suppression mechanism [50]. Another group showed a similar phenomenon in PTEN-deficient prostate tissue, which they interpreted as confirming the activation of a p21-related senescence pathway[25]. However, ours is the first study to report increased p21 levels and altered subcellular compartment localization in PTEN-knockdown cells, and to begin to elucidate a mechanism for this finding.

Our use of a kidney cancer cell line for these studies was prompted by the facts that (i) kidney cancer is exquisitely chemotherapy-resistant such that 5-year survival for the metastatic form of the disease is less than 10% [36], and (ii) decreased PTEN expression is frequently seen in this disease[8,9]. In an earlier study, we found elevated levels of cytoplasmic p21 expression at time of surgery were associated with a worse prognosis in patients with metastatic ccRCC[26] consistent with the data in the present study; assessment of PTEN and p21 status in a small set of ccRCC (N = 9) from the University of Colorado Hospital archives showed loss of PTEN and elevated levels of p21 staining in every tumor, as compared to unaffected kidney samples from age and sex matched controls (R. Weiss, J. Modiano, and K. Shroyer, unpublished observations).

Akt regulates the balance of apoptosis and cell survival by phosphorylating proteins crucial in apoptotic and anti-apoptotic mechanisms. Based on the fact that altered PTEN expression is often observed in RCC, deregulated Akt activation is likely one of the underlying mechanisms of RCC tumorigenesis. Use of the Akt inhibitor of the phosphatidylinositol ether lipid analogue class that we utilized in this study has been shown to cause apoptosis in various RCC lines which are characterized by elevated Akt activation [8]. Among a variety of downstream targets of Akt, phosphorylation of Bad, an important pro-apoptotic protein, was prominently decreased when treated with the Akt inhibitor, possibly due to decreased Akt activity. In addition, expression of Bcl-XL, an anti-apoptotic protein, was decreased resulting in increased cell death. Consistent with these findings, our data suggest that pharmaceutical approaches to target Akt inhibition may be useful in designing more efficient therapeutic regimens for ccRCC.

The most recognized and universal response of a cell to DNA damage is activation of p53 transcription, followed by p53-mediated transcriptional activation. p53, the "guardian of the genome" is responsible for apoptosis of cells which cannot be repaired, and some of these biochemical events are mediated by p21[24]. However, it has been described that PTEN may protect p53 from Mdm-2-mediated degradation[51], so it is possible that the observed increase in p21 levels is mediated via p53 or Mdm-2, or through a p53-independent mechanism.

The clinical relevance of our findings is supported by recent data indicating that p21 is involved in the mechanism of action of mTOR inhibitors. Beuvink et al showed that the mTOR inhibitor RAD001 sensitizes cells to DNA damage-induced apoptosis through inhibition of p21 translation[34]. Our data suggests an explanation for the anti-tumor activity of mTOR inhibition in a clinical trial of RCC[35], especially in those RCCs with PTEN alterations, and these data support further trials of mTOR inhibitors in RCC and other HIF-activated cancers.

One possible means to sensitize cancer cells to DNA damage induced apoptosis would be to attenuate p21 prior to application of a DNA damaging agent in order to direct DNA damaged cells into the apoptotic (rather than the repair) pathway[23,24]. Our reported successful use of phosphorothioated antisense p21 oligonucleotides in several cell lines, including renal mesangial cells [21,23,52,53], could easily be extended to kidney disease *in vivo*, given the high transport of such oligonucleotides to kidney tubular epithelial cells when systemically administered[54]. Experiments to test this possibility using both tissue culture and an animal model of RCC are currently underway in our laboratory.

## Conclusion

In this study, we have found that knockdown of the commonly altered tumor suppressor gene *PTEN *results in increased stability and cytosolic localization of the pleiotropic cell cycle protein p21, both properties being historically associated with resistance to apoptosis. Whether this is a mechanism by which tumors escape chemotherapeutic death is a topic of active investigation in our laboratories; however, our findings suggest the use of novel p21-attenuating methods in combination with conventional chemotherapy in the treatment of chemotherapy-resistant kidney and other cancers.

## Methods

### Chemicals and reagents

The PI3K inhibitors LY-294,002 hydrochloride and wortmannin, and the proteasome inhibitors, N-acetyl-L-leucinyl-L-leucinal-L-norleucinal (LLnL), lactacystin and MG132, were purchased from Sigma-Aldrich (St. Louis, MO). The Akt inhibitor PIA5 was kindly provided by Dr. Phillip Dennis (Medical Oncology Branch, NCI). Anti-Akt, anti-phospho-Akt (Ser473), anti-mono-methyl Histone H3, anti-phospho-MDM2 (Ser 166), and anti-PTEN antibodies were purchased from Cell Signaling Technology (Danvers, MA). Anti-p21 antibody was purchased from Upstate Biotechnology, Inc. (Charlottesville, VA). Anti-GAPDH antibody was from Chemicon-Millipore (Temecula, CA). Anti-phospho-threonine antibody was from Abcam (Cambridge, MA); and phospho-serine detection kit containing four different anti-phospho-serine antibodies, was from Calbiochem (San Diego, CA).

### Design of vectors for RNA inhibition

We used the pSuper platform (OligoEngine, Seattle, WA) to clone PTEN short hairpin RNA molecules (shRNAs) as described[55]. Small inhibitory RNA sequences were designed using a program similar to OligoEngine and Ambion (Austin, TX) online tools. Two sites that were common to both programs were selected, corresponding to positions 1,159 – 1,181 (AAGGCGTATACAG GAACAATATT) and 1,469 – 1,491 (AAGGCACAAGAGGCCCTAGATTT) from the human PTEN mRNA (Genbank accession # NM000314), where the ATG start site is at position 1,032. The oligonucleotides were cloned into the BglII/HindIII sites of the pSuper-puro vector as described [55]. Specificity controls included the empty vector and a double base pair mutant for the 1,469 – 1,491 PTEN shRNA sequence (Fig. 1), where the resulting oligonucleotide did not show any positive matches for any mammalian sequence using basic blast (NCBI, National Library of Medicine, NIH). Stable cell lines integrating PTEN shRNA, mutant PTEN control, or empty pSuper-puro vector were selected in puromycin containing media.

### Cells and cell lines

ACHN human RCC cells (CRL-1161) were obtained from ATCC (Manassas, VA) and maintained in MEM 1X media supplemented with 10% fetal bovine serum, 2 mM L-glutamine, and 1 mM sodium pyruvate. For transfections, 1 × 10^6 ^cells were placed in a sterile cuvette with 4 μg of the corresponding pSuper vector + 1 μg of pEGFP-C vector (BD-Clontech, Mountain View, CA), and transfected using the AMAXA Nucleofector (Amaxa, Gaithersburg, MD) in solution V, program T20 following the manufacturer's instructions for transfection of adherent cells. Transfection efficiency was monitored microscopically based on the number of cells showing green fluorescence. Selection of stable cell lines was accomplished by adding 2.5 μg/ml puromycin to cells 24 hr after transfection. After 5 days, surviving cells were transferred to media containing 1 μg/ml puromycin, and the cells were maintained in that formulation throughout the duration of experiments. Retention of the transfected plasmids was routinely monitored by assessing green fluorescence in cells under selection, and by repeated assessment of PTEN levels using immunoblotting.

### Western blotting

Cells were solubilized in lysis buffer (50 mM HEPES, 1% Triton X-100, 10 mM Na Pyrophosphate, 100 mM Na Fluoride, 4 mM EDTA) and equal protein quantities were electrophoresed and immunoblotted as previously described [21]. The membranes were blocked in 5–10% non-fat dry milk or 1% BSA (for modification-state antibodies) for 1 h at room temperature, and probed with appropriate antibodies. Membranes were then probed with HRP-tagged anti-mouse or anti-rabbit IgG antibodies (Bio-Rad; Amersham) diluted 1:5,000 – 1:15,000 in 2.5–5% non-fat dry milk for 1 h at room temperature. Chemiluminescence was detected using enhanced ECL (Bio-Rad; Amersham Biosciences).

### Immunohistochemistry

Data previously reported in reference[38] were re-evaluated to determine the universality of the relationship between expression and localization of PTEN and p21. Briefly, archival tissues from canine melanomas were identified and 5 μm serial sections from paraffin-embedded blocks were mounted onto positively charged slides (Probe-on, Fisher Scientific, Pittsburgh, PA). Immunostaining was performed using a modified streptavidin- biotin complex method. Antigen retrieval was done by microwave heating for 6 minutes in a buffer of 0.1 M sodium citrate (pH 6). Samples were graded for intensity and percentage of positive cells, as well as for subcellular location of the relevant stain. Samples were considered negative when no staining was seen above that seen in negative controls. Staining that was fine, diffuse, and not always visible at low magnification (40–100-X), but was readily detectable in >30% of cells at high magnification (4,000-X) was considered weak. Staining that was prominent enough to be seen at low magnification in >30% of cells (and was verifiable as specific staining at high magnification) was considered moderate to strong. Samples were only considered positive when expression of these proteins was clearly detectable in melanocytic tumor cells (versus supporting stroma or inflammatory cells) based on the expression of S100a protein, Melan A, or neuron-specific enolase in serial sections from the tumors.

### Protein stability assays

Approximately 2.5 × 10^5 ^cells were grown on 6-well tissue culture dishes in media containing 10% fetal bovine serum. Cells were treated with 20 μg/ml cycloheximide (Sigma-Aldrich) at 30 min interval up to a maximum of 2.5 h. Cells were collected and analyzed by immunoblotting.

### Immunofluorescence microscopy

Cells were grown on Lab-Tek multi-chamber slides (Nalge Nunc International, Rochester; NY, USA) and fixed in cold methanol for 10 min at -20°C. Fixed cells were rehydrated with PBS and blocked with 10% goat serum (Jackson ImmunoResearch Laboratories, Inc.) for 1 h at room temperature. Cells were incubated with anti-p21 antibody (1:50) for 30 min at room temperature, followed by a secondary goat anti-mouse IgG antibody (1:1000) conjugated to Alexa-Fluor 594 (Molecular Probes) for 30 min at room temperature. DAPI (0.1 μg/ml, 5 min at room temperature) was used for nuclear counterstaining. The LSM 5 Pascal confocal laser scanning microscope was used for imaging (Carl Zeiss; Thornwood, NY, USA).

### Caspase assay

The CaspACE assay kit (Promega, Madison, WI) was used to measure caspase-3 activity per the manufacturer's instructions. Briefly, 10^6 ^cells per 10 cm dish were treated as described in the text. Positive control cells were treated with 4 μM camptothecin. The cells were harvested, washed, and equal protein quantities were incubated with the DEVD-pNA caspace-3 substrate in Caspase Assay Buffer. The plates were covered with parafilm and incubated at 37°C overnight. Color development was measured at 405 nm.

### Subcellular fractionation

Cells were grown in 100 cm dishes to 95% confluency followed by separation into nuclear and cytosolic fractions using the Panomics Nucelar extraction kit (Panomics, Fremont, CA) according to the manufacturer's instructions. Briefly, cells were washed with PBS twice and lysed in a buffer containing 100 mM HEPES, pH 7.9, 100 mM KCl, and 100 mM EDTA on ice for 10 minutes. Lysates were centrifuged at 15,000 × g for 3 min at 4°C to obtain cytosolic extracts. Soluble nuclear extracts were obtained by vortexing nuclear pellets in a buffer containing 100 mM HEPES, pH 7.9, 2 M NaCl, 5 mM EDTA, 50% glycerol, protease inhibitor cocktail and 100 mM DTT for 10 sec and then centrifuged at 15,000 × g for 5 min at 4°C. Protein concentrations in each fraction were determined using the BioRad protein assay. Relative enrichment of the cytosolic and nuclear fractions, respectively, were determined by immunoblotting with antibodies against GAPDH and Mono-Methyl Histone H3.

## Competing interests

The author(s) declare that they have no competing interests.

## Authors' contributions

RHW conceived of the study and wrote the original and final versions of the manuscript. JFM helped design all experiments and assisted with the initial concept, experimental design, and all drafts of the manuscript. SB provided the siRNA constructs. PYL and SPF performed all of the experiments with assistance from SHP and JYP and contributed to writing of each draft of the manuscript.

## References

[B1] Li J, Yen C, Liaw D, Podsypanina K, Bose S, Wang SI, Puc J, Miliaresis C, Rodgers L, McCombie R, Bigner SH, Giovanella BC, Ittmann M, Tycko B, Hibshoosh H, Wigler MH, Parsons R (1997). PTEN, a putative protein tyrosine phosphatase gene mutated in human brain, breast, and prostate cancer. Science.

[B2] Stambolic V, Tsao MS, Macpherson D, Suzuki A, Chapman WB, Mak TW (2000). High incidence of breast and endometrial neoplasia resembling human Cowden syndrome in pten+/- mice. Cancer Res.

[B3] Podsypanina K, Ellenson LH, Nemes A, Gu J, Tamura M, Yamada KM, Cordon-Cardo C, Catoretti G, Fisher PE, Parsons R (1999). Mutation of Pten/Mmac1 in mice causes neoplasia in multiple organ systems. Proc Natl Acad Sci U S A.

[B4] Wang S, Gao J, Lei Q, Rozengurt N, Pritchard C, Jiao J, Thomas GV, Li G, Roy-Burman P, Nelson PS, Liu X, Wu H (2003). Prostate-specific deletion of the murine Pten tumor suppressor gene leads to metastatic prostate cancer. Cancer Cell.

[B5] Yilmaz OH, Valdez R, Theisen BK, Guo W, Ferguson DO, Wu H, Morrison SJ (2006). Pten dependence distinguishes haematopoietic stem cells from leukaemia-initiating cells. Nature.

[B6] Marsh DJ, Coulon V, Lunetta KL, Rocca-Serra P, Dahia PL, Zheng Z, Liaw D, Caron S, Duboue B, Lin AY, Richardson AL, Bonnetblanc JM, Bressieux JM, Cabarrot-Moreau A, Chompret A, Demange L, Eeles RA, Yahanda AM, Fearon ER, Fricker JP, Gorlin RJ, Hodgson SV, Huson S, Lacombe D, Eng C, . (1998). Mutation spectrum and genotype-phenotype analyses in Cowden disease and Bannayan-Zonana syndrome, two hamartoma syndromes with germline PTEN mutation. Hum Mol Genet.

[B7] Lee JS, Kim HS, Kim YB, Lee MC, Park CS (2003). Expression of PTEN in renal cell carcinoma and its relation to tumor behavior and growth. J Surg Oncol.

[B8] Hara S, Oya M, Mizuno R, Horiguchi A, Marumo K, Murai M (2005). Akt activation in renal cell carcinoma: contribution of a decreased PTEN expression and the induction of apoptosis by an Akt inhibitor. Ann Oncol.

[B9] Brenner W, Farber G, Herget T, Lehr HA, Hengstler JG, Thuroff JW (2002). Loss of tumor suppressor protein PTEN during renal carcinogenesis. Int J Cancer.

[B10] Alimov A, Li C, Gizatullin R, Fredriksson V, Sundelin B, Klein G, Zabarovsky E, Bergerheim U (1999). Somatic mutation and homozygous deletion of PTEN/MMAC1 gene of 10q23 in renal cell carcinoma. Anticancer Res.

[B11] Sukosd F, Digon B, Fischer J, Pietsch T, Kovacs G (2001). Allelic loss at 10q23.3 but lack of mutation of PTEN/MMAC1 in chromophobe renal cell carcinoma. Cancer Genet Cytogenet.

[B12] Figlin RA, Seligson D, Wu H, Thomas G, Leppert JT, O'Toole T, Dukart G, Gibbons J, Belldegrun A, Pantuck AJ (2005). Characterization of the mTOR pathway in renal cell carcinoma and its use in predicting patient selection for agents targeting this pathway. J Clin Oncol.

[B13] Eng C (2003). PTEN: one gene, many syndromes. Hum Mutat.

[B14] Zhou M, Gu L, Findley HW, Jiang R, Woods WG (2003). PTEN reverses MDM2-mediated chemotherapy resistance by interacting with p53 in acute lymphoblastic leukemia cells. Cancer Res.

[B15] Wendel HG, Malina A, Zhao Z, Zender L, Kogan SC, Cordon-Cardo C, Pelletier J, Lowe SW (2006). Determinants of sensitivity and resistance to rapamycin-chemotherapy drug combinations in vivo. Cancer Res.

[B16] Sherr CJ, Roberts JM (1999). CDK inhibitors: positive and negative regulators of G1-phase progression. Genes and Dev.

[B17] Xiong Y, Hannon GJ, Zhang H, Casso D, Kobayashi R, Beach D (1993). p21 is a universal inhibitor of cyclin kinases. Nature.

[B18] Harper JW, Adami GR, Wei N, Keyomarsi K, Elledge SJ (1993). The p21 Cdk-interacting protein Cip1 is a potent inhibitor of G1 cyclin-dependent kinases. Cell.

[B19] LaBaer J, Garrett MD, Stevenson LF, Slingerland JM, Sandhu C, Chou HS, Fattaey A, Harlow E (1997). New functional activities for the p21 family of CDK inhibitors. Genes Dev.

[B20] Cheng M, Sexl V, Sherr CJ, Roussel MF (1998). Assembly of cyclin D-dependent kinase and titration of p27Kip1 regulated by mitogen-activated protein kinase kinase (MEK1). Proc Natl Acad Sci U S A.

[B21] Weiss RH, Joo A, Randour C (2000). p21Waf1/Cip1 is an assembly factor required for PDGF-induced vascular smooth muscle cell proliferation. J Biol Chem.

[B22] Weiss RH, Marshall D, Howard L, Corbacho AM, Cheung AT, Sawai ET (2003). Suppression of breast cancer growth and angiogenesis by an antisense oligodeoxynucleotide to p21(Waf1/Cip1). Cancer Lett.

[B23] Fan Y, Borowsky AD, Weiss RH (2003). An antisense oligodeoxynucleotide to p21(Waf1/Cip1) causes apoptosis in human breast cancer cells. Mol Cancer Ther.

[B24] Weiss RH (2003). p21Waf1/Cip1 as a therapeutic target in breast and other cancers. Cancer Cell.

[B25] Chen Z, Trotman LC, Shaffer D, Lin HK, Dotan ZA, Niki M, Koutcher JA, Scher HI, Ludwig T, Gerald W, Cordon-Cardo C, Pandolfi PP (2005). Crucial role of p53-dependent cellular senescence in suppression of Pten-deficient tumorigenesis. Nature.

[B26] Weiss RH, Borowsky AD, Seligson D, Lin PY, Dillard-Telm L, Belldegrun AS, Figlin RA, Pantuck AD (2007). p21 is a prognostic marker for renal cell carcinoma: implications for novel therapeutic approaches. J Urol.

[B27] Perroud B, Lee J, Valkova N, Dhirapong A, Lin PY, Fiehn O, Kultz D, Weiss RH (2006). Pathway analysis of kidney cancer using proteomics and metabolic profiling. Mol Cancer.

[B28] Li Y, Jenkins CW, Nichols MA, Xiong Y (1994). Cell cycle expression and p53 regulation of the cyclin-dependent kinase inhibitor p21. Oncogene.

[B29] Li Y, Dowbenko D, Lasky LA (2002). AKT/PKB phosphorylation of p21Cip1/WAF1 enhances protein stability of p21Cip1/WAF1 and promotes cell survival. J Biol Chem.

[B30] Zhou BP, Liao Y, Xia W, Spohn B, Lee MH, Hung MC (2001). Cytoplasmic localization of p21Cip1/WAF1 by Akt-induced phosphorylation in HER-2/neu-overexpressing cells.. Nat Cell Biol.

[B31] Ritt MG, Mayor J, Wojcieszyn J, Smith R, Barton CL, Modiano JF (2000). Sustained nuclear localization of p21/WAF-1 upon growth arrest induced by contact inhibition. Cancer Lett.

[B32] Dong Y, Chi SL, Borowsky AD, Fan Y, Weiss RH (2003). Cytosolic p21Waf1/Cip1 increases cell cycle transit in vascular smooth muscle cells. Cell Signal.

[B33] Asada M, Yamada T, Ichijo H, Delia D, Miyazono K, Fukumuro K, Mizutani S (1999). Apoptosis inhibitory activity of cytoplasmic p21(Cip1/WAF1) in monocytic differentiation. EMBO J.

[B34] Beuvink I, Boulay A, Fumagalli S, Zilbermann F, Ruetz S, O'Reilly T, Natt F, Hall J, Lane HA, Thomas G (2005). The mTOR inhibitor RAD001 sensitizes tumor cells to DNA-damage-induced apoptosis through inhibition of p21 translation. Cell.

[B35] Atkins MB, Hidalgo M, Stadler WM, Logan TF, Dutcher JP, Hudes GR, Park Y, Liou SH, Marshall B, Boni JP, Dukart G, Sherman ML (2004). Randomized phase II study of multiple dose levels of CCI-779, a novel mammalian target of rapamycin kinase inhibitor, in patients with advanced refractory renal cell carcinoma. J Clin Oncol.

[B36] Weiss RH, Lin PY (2006). Kidney Cancer: Identification of Novel Targets for Therapy. Kidney Int.

[B37] Nanus DM, Garino A, Milowsky MI, Larkin M, Dutcher JP (2004). Active chemotherapy for sarcomatoid and rapidly progressing renal cell carcinoma. Cancer.

[B38] Koenig A, Bianco SR, Fosmire S, Wojcieszyn J, Modiano JF (2002). Expression and significance of p53, rb, p21/waf-1, p16/ink-4a, and PTEN tumor suppressors in canine melanoma. Vet Pathol.

[B39] Rossig L, Jadidi AS, Urbich C, Badorff C, Zeiher AM, Dimmeler S (2001). Akt-dependent phosphorylation of p21Cip1 regulates PCNA binding and proliferation in endothelial cells. Mol Cell Biol.

[B40] Bloom J, Amador V, Bartolini F, DeMartino G, Pagano M (2003). Proteasome-mediated degradation of p21 via N-terminal ubiquitinylation. Cell.

[B41] Rousseau D, Cannella D, Boulaire J, Fitzgerald P, Fotedar A, Fotedar R (1999). Growth inhibition by CDK-cyclin and PCNA binding domains of p21 occurs by distinct mechanisms and is regulated by ubiquitin-proteasome pathway. Oncogene.

[B42] Blagosklonny MV, Wu GS, Omura S, el Deiry WS (1996). Proteasome-dependent regulation of p21WAF1/CIP1 expression. Biochem Biophys Res Commun.

[B43] Raftopoulou M, Etienne-Manneville S, Self A, Nicholls S, Hall A (2004). Regulation of cell migration by the C2 domain of the tumor suppressor PTEN. Science.

[B44] Gills JJ, Holbeck S, Hollingshead M, Hewitt SM, Kozikowski AP, Dennis PA (2006). Spectrum of activity and molecular correlates of response to phosphatidylinositol ether lipid analogues, novel lipid-based inhibitors of Akt. Mol Cancer Ther.

[B45] Winters ZE, Leek RD, Bradburn MJ, Norbury CJ, Harris AL (2003). Cytoplasmic p21Waf1/Cip1 expression is correlated with HER-2/neu in breast cancer and is an independent predictor of prognosis. Breast Cancer Res.

[B46] Winters ZE, Hunt NC, Bradburn MJ, Royds JA, Turley H, Harris AL, Norbury CJ (2001). Subcellular localisation of cyclin B, Cdc2 and p21(WAF1/CIP1) in breast cancer. association with prognosis. Eur J Cancer.

[B47] Kastan MB, Onyekwere O, Sidransky D, Vogelstein B, Craig RW (1991). Participation of p53 protein in the cellular response to DNA damage. Cancer Res.

[B48] Kuerbitz SJ, Plunkett BS, Walsh WV, Kastan MB (1992). Wild-type p53 is a cell cycle checkpoint determinant following irradiation. Proc Natl Acad Sci U S A.

[B49] Wu H, Goel V, Haluska FG (2003). PTEN signaling pathways in melanoma. Oncogene.

[B50] Yoo LI, Liu DW, Le Vu S, Bronson RT, Wu H, Yuan J (2006). Pten deficiency activates distinct downstream signaling pathways in a tissue-specific manner. Cancer Res.

[B51] Freeman DJ, Li AG, Wei G, Li HH, Kertesz N, Lesche R, Whale AD, Martinez-Diaz H, Rozengurt N, Cardiff RD, Liu X, Wu H (2003). PTEN tumor suppressor regulates p53 protein levels and activity through phosphatase-dependent and -independent mechanisms. Cancer Cell.

[B52] Weiss RH, Randour CJ (2002). Attenuation of matrix protein secretion by antisense oligodeoxynucleotides to the cyclin kinase inhibitor p21(Waf1/Cip1). Atherosclerosis.

[B53] Fan YP, Weiss RH (2004). Exogenous attenuation of p21(Waf1/Cip1) decreases mesangial cell hypertrophy as a result of hyperglycemia and IGF-1. J Am Soc Nephrol.

[B54] Rappaport J, Hanss B, Kopp JB, Copeland TD, Bruggeman LA, Coffman TM, Klotman PE (1995). Transport of phosphorothioate oligonucleotides in kidney: implications for molecular therapy. Kidney Int.

[B55] Baksh S, Tommasi S, Fenton S, Yu VC, Martins LM, Pfeifer GP, Latif F, Downward J, Neel BG (2005). The tumor suppressor RASSF1A and MAP-1 link death receptor signaling to Bax conformational change and cell death. Mol Cell.

